# Functional morphology and integration of corvid skulls – a 3D geometric morphometric approach

**DOI:** 10.1186/1742-9994-6-2

**Published:** 2009-01-07

**Authors:** Christoph Kulemeyer, Kolja Asbahr, Philipp Gunz, Sylke Frahnert, Franz Bairlein

**Affiliations:** 1 Institut fur Vogelforschung, "Vogelwarte Helgoland", An der Vogelwarte 21, 26386 Wilhelmshaven, Germany; 2 Museum fur Naturkunde – Leibniz-Institut für Evolutions- und Biodiversitätsforschung an der Humboldt-Universität zu Berlin, Invalidenstr 43, 10115 Berlin, Germany; 3 Department of Human Evolution, Max-Planck Institute for Evolutionary Anthropology, Deutscher Platz 6, 04103 Leipzig, Germany

## Abstract

**Background:**

Sympatric corvid species have evolved differences in nesting, habitat choice, diet and foraging. Differences in the frequency with which corvid species use their repertoire of feeding techniques is expected to covary with bill-shape and with the frontal binocular field. Species that frequently probe are expected to have a relatively longer bill and more sidewise oriented orbits in contrast to species that frequently peck. We tested this prediction by analyzing computed tomography scans of skulls of six corvid species by means of three-dimensional geometric morphometrics. We (1) explored patterns of major variation using principal component analysis, (2) compared within and between species relationships of size and shape and (3) quantitatively compared patterns of morphological integration between bill and cranium by means of partial least squares (singular warp) analysis.

**Results:**

Major shape variation occurs at the bill, in the orientation of orbits, in the position of the foramen magnum and in the angle between bill and cranium. The first principal component correlated positively with centroid-size, but within-species allometric relationships differed markedly. Major covariation between the bill and cranium lies in the difference in orbit orientation relative to bill-length and in the angle between bill and cranium.

**Conclusion:**

Corvid species show pronounced differences in skull shape, which covary with foraging mode. Increasing bill-length, bill-curvature and sidewise orientation of the eyes is associated with an increase in the observed frequency in probing (vice versa in pecking). Hence, the frequency of probing, bill-length, bill-curvature and sidewise orientation of the eyes is progressively increased from jackdaw, to Eurasian jay, to black-billed magpie, to hooded crow, to rook and to common raven (when feeding on carcasses is considered as probing). Our results on the morphological integration suggest that most of the covariation between bill and cranium is due to differences in the topography of the binocular fields and the projection of the bill-tip therein, indicating the importance of visual fields to the foraging ecology of corvids.

## Background

Sympatric corvids in Central Europe have evolved differences in nesting [1-3], habitat choice [3-5], diet [1,4,6] and foraging [4-7]. Although these species overlap in their feeding techniques, the quantity with which they use their repertoire is markedly different between species. In behavioral studies, three main feeding techniques have been defined: (1) probing, which is characterized by feeding below the surface, (2) pecking, which is characterized by feeding at the surface and (3) turning objects, which represents searching and feeding beneath animal dung and other surface litter [4-8]. Probing is frequently observed in rook, hooded crow and common raven, while pecking and turning objects is often found in Eurasian jay, jackdaw, black-billed magpie and hooded crow [4-7,9,10]. It is assumed that foraging behavior covaries with bill morphology [11-14] and that probing is observed more frequently in birds with long and curved bills, while pecking and turning objects are observed more frequently in birds with straight and short bills [15-18].

While bill morphology has been studied intensively, its covariation with other components of the avian skull has hardly been considered and never been quantified (e.g. [16,19-21]). For instance, the adaptive significance of the frontal binocular field in foraging ecology is well known in birds [22,23]. Thus, most of the variation in the topography of binocular fields, its width, vertical extent and the horizontal and vertical projection of the bill-tip within the binocular field, have been explained by differences in foraging behavior [22,23]. Hence, probing in contrast to pecking and turning objects is thought to require a smaller frontal binocular field, because its principal function lies in the degree to which vision is used in the guidance of the bill towards food objects [22,23]. In morphological studies, several authors assumed that orbit convergence, i. e. the orbit orientation in skulls, is associated with the degree of the binocular field overlap [23-25].

In this paper, we analyzed computed tomography (CT) scans of skulls of six corvid species by means of three-dimensional geometric morphometrics to (1) explore patterns of major variation using principal component analysis, (2) compare within and between species relationships of size and shape and (3) quantitatively compare patterns of morphological integration between bill and cranium. We expect corvid species that frequently probe to have a longer and more curved bill and more sidewise oriented orbits, when compared with corvids, which frequently peck and turn objects.

## Methods

### Data

Our sample consists of 115 adult skulls of six corvid species: common raven (*Corvus corax*), hooded crow (*Corvus corone cornix*), rook (*Corvus frugilegus*), jackdaw (*Corvus monedula*), black-billed magpie (*Pica pica*) and Eurasian jay (*Garrulus glandarius*). The specimens are almost equally distributed across species and came from Museum für Naturkunde Berlin, Staatliches Museum für Naturkunde Görlitz and Staatssammlung für Anthropologie und Paläoanatomie München, which are all located in Germany. All corvid skulls were scanned by computed tomography at Charite-Universitätsmedizin Berlin.

**Figure 1:**
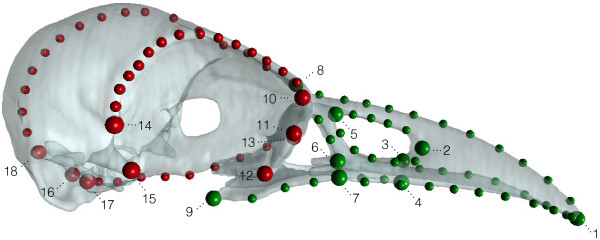
**Landmarks and semilandmarks**. Lateral view of a corvid skull with landmarks (big dots) and semilandmarks (small dots) partitioned into two blocks: bill (green) and cranium (red).

We digitized three-dimensional coordinates of 32 homologous landmarks on evolutionary stable structures and 116 equally spaced semilandmarks on 16 curves (Tab. 1, Fig. 1). Semilandmarks refer to a series of points that are sampled along outlines and that are allowed to slide along curves to minimize bending energy. In subsequent statistical analysis, these relaxed semilandmarks can be treated as homologous within the sample [26-28]. Digitization of landmarks and semilandmarks, as well as the processing of semilandmarks were done with Edgewarp 3.30 [29].

**Table 1:** Landmark description.

Number	Description
bill	86 Landmarks and semilandmarks

1	bill-tip
2 L, R	maximum of curvature at the rostral end of the external nares
3	most caudal point of the ossified palatine
4 L, R	bifurcation of the Os palatinum and the Os maxillare close to the rim of the Os maxillare
5 L, R	maximum of curvature at the caudal end of the external nares
6 L, R	maximum of curvature at the rostral end of the Fossa et Fenestra antorbitalis
7 L, R	maximum of curvature at the lateral intersection of the Processus maxillaris and the Os jugale
8	mid-point of the cranio-facial hinge
9 L, R	most posterior point of the Angulus caudolatum (Os palatinum)

cranium	66 Landmarks and semilandmarks

10 L, R	most dorso-lateral point of the Os lacrimale
11 L, R	most medial point (maximum of curvature) of the Os lacrimale
12 L, R	most ventro-lateral point of the Os lacrimale
13	most ventro-rostral point of the Os mesethmoidale
14 L, R	most distal point of the Processus postorbitalis
15 L, R	most distal point of the Processus zygomaticus
16	most caudal point of the Condylus occipitalis
17 L, R	Ostium canalis ophthalmici externi
18	most dorsal point of the Foramen magnum

Descriptions of landmarks [59] that were digitized on two blocks: bill and cranium; either placed on the midsagittale or on the left and right (L, R)

### Geometric morphometrics

The resulting dataset was subjected to a generalized least squares Procrustes analysis (GPA, [30]), in which distances between homologous landmarks are minimized by translating, rotating and scaling all forms to a common reference (consensus). In other words, shape refers to the geometric information that is left after removing the effects of size, position and orientation. The information about overall body-size of the specimens is preserved in centroid size, which serves as a scaling factor in GPA and which is calculated as the square root of the sum of squared distances of landmarks and semilandmarks from their centroid.

In birds, especially in those with long bills, landmarks and semilandmarks that are placed closely to the bill-tip will have a great influence on centroid size, because they change the position of the centroid and thus change the distance of the whole landmark set to its centroid. Bills are known to vary greatly according to foraging mode. Hence, centroid sizes calculated from the whole landmark set, i.e. including the bill, will not be a good predictor of overall body-size if bill-length in the studied species is highly variable. Therefore, we adjusted GPA by using centroid size calculated only from landmarks and semilandmarks placed on the cranium and the antorbital fenestra: landmarks 6 – 18 and associated semilandmarks (Fig. 1, Tab. 1).

Procrustes shape coordinates, returned by GPA, were subjected to a principal component analysis (PCA) to explore patterns of major variation across the entire skull. To test the influence of size on shape, a multivariate linear regression of the Procrustes shape coordinates on log centroid size was performed.

### Partial least squares

Partial least squares analysis (PLS) is often referred to as singular warp analysis when applied to Procrustes shape coordinates [31-33]. PLS is used to find correlated pairs of linear combinations between two blocks of variables. These linear combinations are constructed to display patterns of covariation between the two blocks, just as PCA extracts patterns of variation across the entire block.

To study the morphological integration between bill and cranium, the Procrustes shape coordinates were partitioned prior to the PLS into these functional units (Fig. 1), which we refer to as the "*whole fit*" dataset. In addition, landmark coordinates of these functional units were scaled and superimposed separately, which we refer to as the "*separate fit*" dataset. In the *whole fit *dataset, the functional units retain their relative position and their size proportions to each other, whereas this geometric information is lost in the *separate fit *dataset. Hence, the analysis of the *separate fit *dataset concentrates purely on differences in shape.

As we are interested whether there are within-group patterns of integration that are shared across species, we standardized the *whole fit *and the *separate fit *dataset by subtracting the species mean from the shape coordinates of the corresponding specimens. These Procrustes residuals were subjected to two PLS, one on the residual shape coordinates of the *whole fit *(PLS_*whole*_) and one on the residual shape coordinates of the *separate fit *dataset (PLS_*sep*_). We used the algorithm introduced by Mitteroecker and Bookstein [34,35] (scaled PLS), which allows a separate scaling of the PLS vectors. This ensures that the amount of shape deformation is correctly scaled when we visualize the shape changes of the two blocks together in the PLS_*whole *_analysis.

All computations were done in Mathematica 6.0 and R 2.6.1. The surface representations of the shape deformations were rendered in Amira 4.0.

## Results

### Species differences

**Figure 2:**
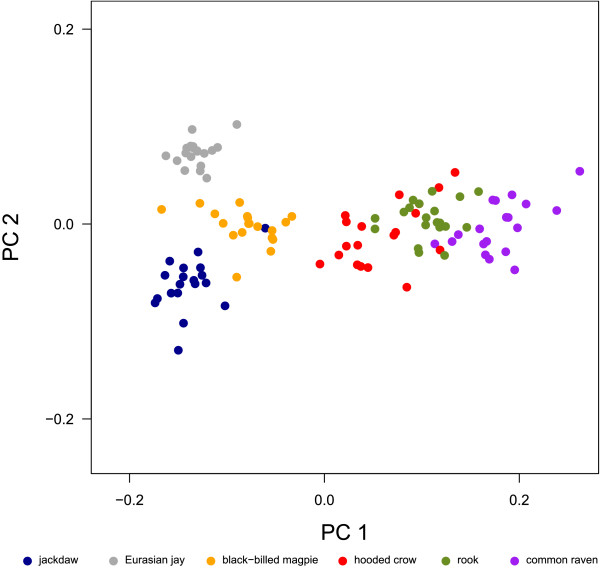
**First two dimensions of the PC scores**. Two-dimensional plot of the principal component scores calculated from the Procrustes shape coordinates of the full landmark set.

**Figure 3:**
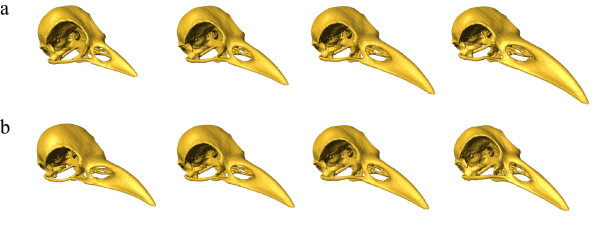
**Shape deformations according to the first two dimensions of the PCA**. The sequence of surface representations (from left to right) as deformations of the average shape correspond to increasing scores in PC 1 **(a) **and PC 2 **(b) **in Figure 2. The surface morphs differ from its neighbors by equal multiples of the standard deviation of the actual variability. The first and last column is extrapolated by 2 **(a) **and 4 **(b) **standard deviations, respectively. **(a): **Major variation in PC 1 lies in the relative length and curvature of the bill, in cranium height, in the position of the foramen magnum and in the position and orientation of the orbits. **(b): **Major variation in PC 2 lies in the angle between bill and cranium, in the position of the foramen magnum, in the position and orientation of the orbits and in bill-curvature.

The PCA of the full landmark set clearly (except from hooded crow and rook) separates the corvid species, which only marginally overlap in the first two principal components (PC, Fig. 2). PC 1 explained 73.7% of the total variation and was interpreted as an increase in relative bill-length, -width and -curvature, an increase in relative cranium length and -width, a decrease in relative cranium height, as an upward positioned foramen magnum and as sidewise oriented orbits (Fig. 3a). PC 2 explained 9.5% of the total variation and was interpreted as an increase in the angle between bill and cranium, which results from a rotation of the cranium. In addition, PC 2 was interpreted as an increase in the curvature of the bill, a decrease in the relative width of bill and cranium, a downward positioned foramen magnum and upward and sidewise oriented orbits (Fig. 3b).

**Figure 4:**
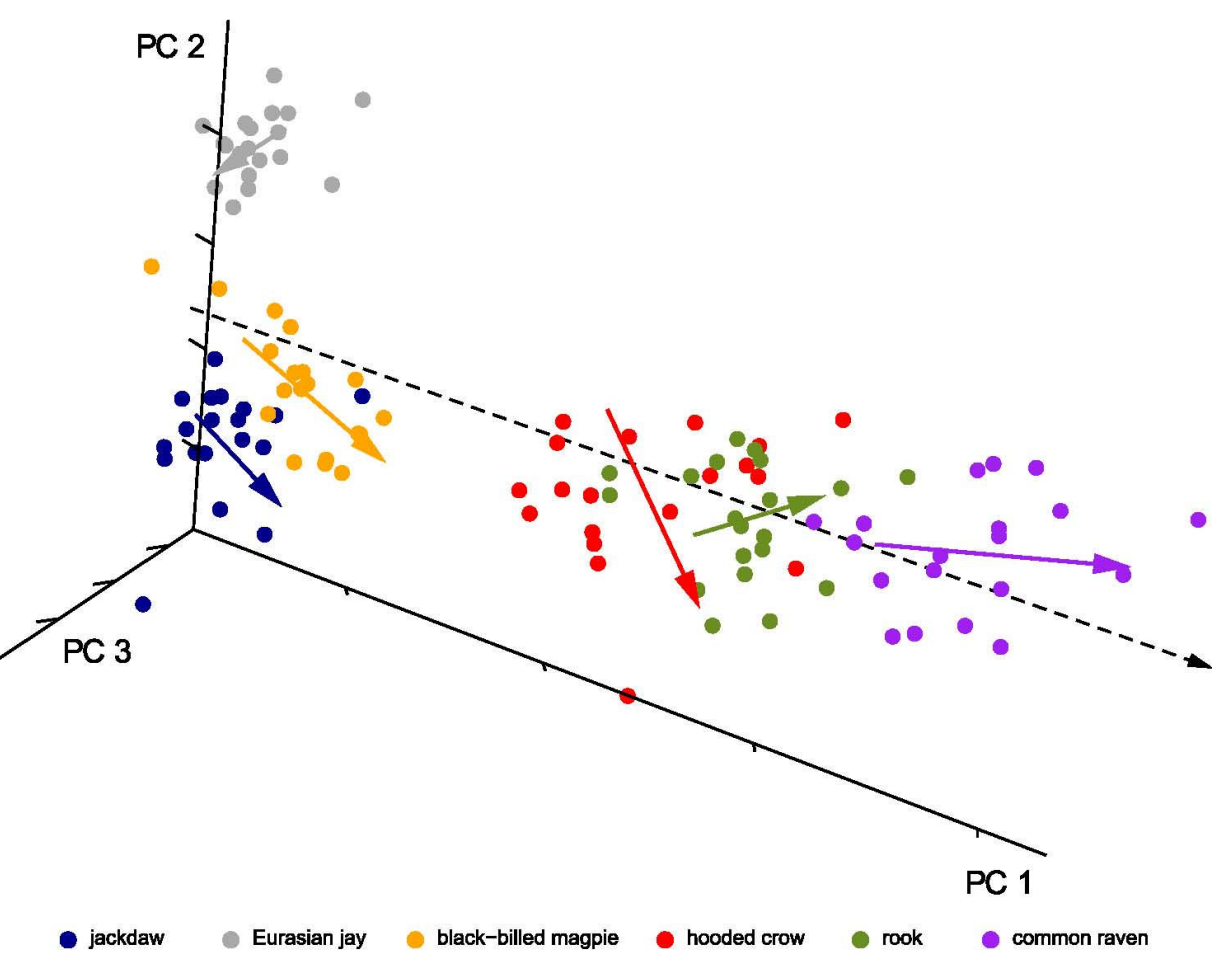
**Multivariate regression of shape variables on log centroid size**. Three-dimensional plot of the principal component scores. The colored lines are intraspecific regressions of the shape coordinates on log centroid size and thus estimate allometry within species. The dashed line is the regression of shape coordinates on log centroid size over all species.

While the scores of PC 1 correlate positively with the log of centroid size (r = 0.927, Fig. 4) and thus reflect shape changes that are associated with differences in size, the scores of PC2 do not correlate with log centroid size (r = -0.08). Note that although rooks have higher scores in PC 1 than hooded crows, centroid size between these two species does not differ (ANOVA, p = 0.959). Thus, shape differences between rooks and hooded crows are not allometric. To explore the influence of size on skull shape, a multivariate linear regression was performed. The correlation between the vector of regression slopes and the first eigenvector of the PCA is very high (r = 0.998, Fig. 4), so that shape changes predicted by the multivariate regression with increasing centroid size resembles shape changes along PC 1. We also regressed the Procrustes shape coordinates on log centroid size for each species separately (Fig. 4). Figure 4 clearly shows that the relationship of size and shape differ markedly between species and thus from the regression vector over all species. As the number of studied individuals per species is small, shape changes that occur with increasing centroid size within species are not visualized.

### Morphological integration

#### Whole fit

**Figure 5:**
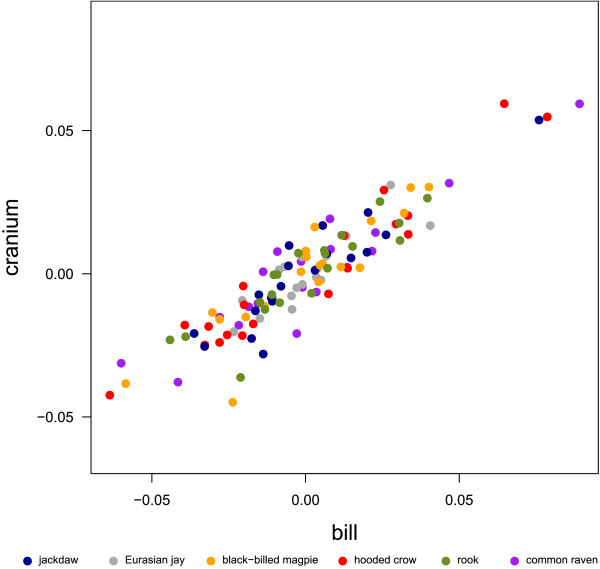
**First dimension of the PLS_*whole *_scores**. First dimension of the partial least squares scores calculated from two landmark blocks, bill and cranium, of the *whole fit *dataset.

**Figure 6:**
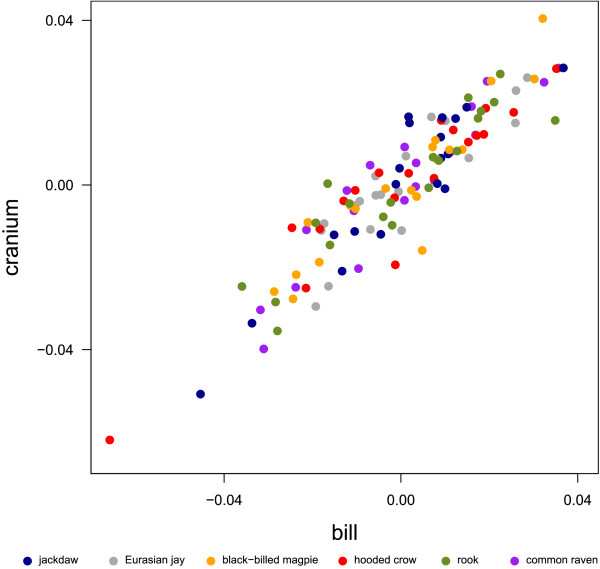
**Second dimension of the PLS_*whole *_scores**. Second dimension of the partial least squares scores calculated from two landmark blocks, bill and cranium, of the *whole fit *dataset.

**Figure 7:**
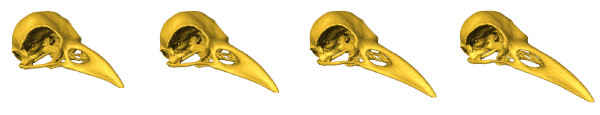
**Shape deformations according to the first PLS_*whole *_dimension**. The sequence of surface representations (from left to right) as deformations of the average shape correspond to increasing scores of the first PLS_*whole *_dimension in Figure 5. The surface morphs differ from its neighbors by equal multiples of the standard deviation of the actual variability. The first and last column is extrapolated by 4 standard deviations. Major covariation between blocks lies in bill- and cranium-length and in the orientation of the eyes.

**Figure 8:**
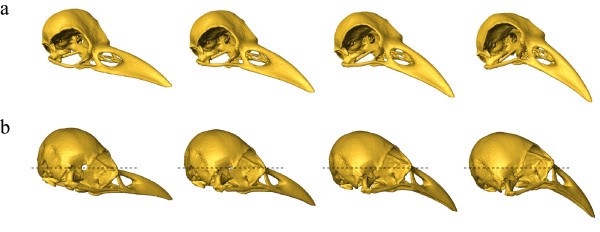
**Shape deformations according to the second PLS_*whole *_dimension**. The sequence of surface representations (from left to right) as deformations of the average shape correspond to increasing scores of the second PLS_*whole *_dimension in Figure 6. The surface morphs differ from its neighbors by equal multiples of the standard deviation of the actual variability. The first and last column is extrapolated by 4 standard deviations. Major covariation between blocks lies in the angle between bill and cranium and in the position of the foramen magnum. **(b): **Exemplary representation of the positional change of the foramen magnum. The dashed line represents the dorsal margin of the foramen magnum of the consensus.

To assess the covariation between the bill and cranium, we performed a PLS analysis on the residual shape coordinates of the *whole fit *dataset, in which the entire landmark set was subjected to one Procrustes fit. The relationship between the functional units is plotted as scores returned by PLS_*whole *_in Figure 5 and 6. These graphs illustrate how well shape and relative position of one block is predicted by shape and relative position of the other (and vice versa). The PLS_*whole *_vectors were visualized as surface deformations in Figure 7 and 8. The correlation between the scores of the first dimension is strikingly high (r = 0.923, Fig. 5) and represents the direction of integration shared by all species. Shape changes of PLS_*whole *_1, visualized in Figure 7, represent the difference between individuals with a long bill and cranium, an upward positioned foramen magnum as well as sidewise oriented orbits and individuals with a short bill and cranium, a downward positioned foramen magnum and forward oriented orbits.

The correlation between scores of the second dimension is very strong (r = 0.922, Fig. 6). Again, the pattern of integration is shared across all species. Major shape changes of PLS_*whole *_2 were interpreted as an increase in the angle between bill and cranium, which results from a rotation of the cranium. These deformations are associated with an increase in bill-curvature, an upward positioned foramen magnum as well as a bending and thus positional adjustment of the palatinum relative to the sphenoidale. Hence, the second dimension represents differences between individuals with a decreased angle between bill and cranium, a curved bill and a more dorsal foramen magnum and individuals with an increased angle between bill and cranium, a straight bill and a more ventral foramen magnum (Fig. 8).

#### Separate fit

**Figure 9:**
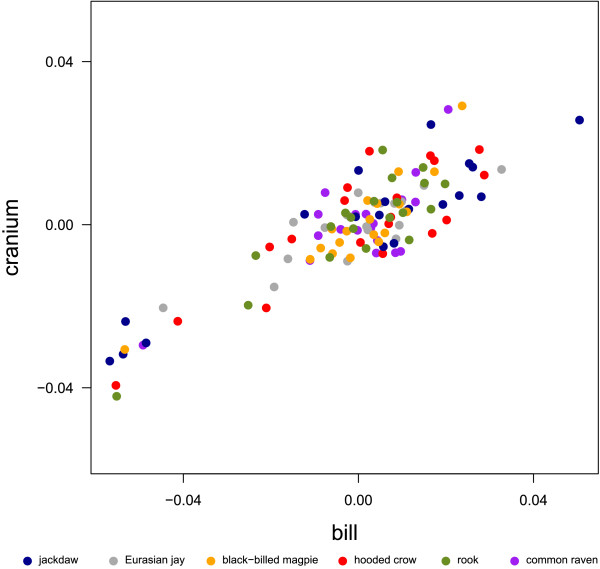
**First dimension of the PLS_*sep *_scores**. First dimension of the partial least squares scores calculated from two landmark blocks, bill and cranium, of the *separate fit *dataset.

**Figure 10:**
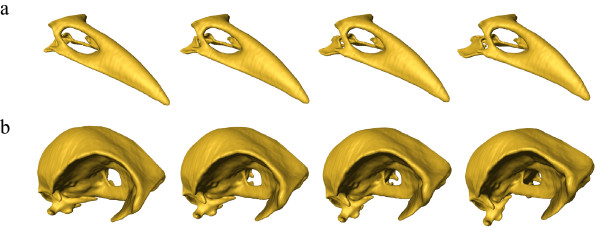
**Shape deformations according to the first PLS_*sep *_dimension**. The sequence of surface representations (from left to right) as deformations of the average shape of the two blocks, bill **(a) **and cranium **(b)**, correspond to increasing scores of the first PLS_*sep *_dimension in Figure 9. The surface morphs differ from its neighbors by equal multiples of the standard deviation of the actual variability. The first and last column is extrapolated by 4 standard deviations. Major covariation between blocks lies in relative bill- and palatinum length **(a) **and in the relative position of the cranio-facial hinge **(b)**.

The covariation independent of the relative position between the bill and cranium was explored by a PLS analysis on the residual shape coordinates of the *separate fit *dataset, in which the two blocks were subjected to separate Procrustes fits. The correlation between the scores of the first PLS_*sep *_vector is strong (r = 0.867, Fig. 9) and the corresponding shape changes (Fig. 10) resemble shape changes displayed by the third dimension of PLS_*whole *_(r = 0.915, not visualized). These differences in shape represent individuals with a long, straight bill, a decreased relative palatinum length and a downward positioned cranio-facial hinge as well as downward positioned orbits, in contrast to individuals with a short, curved bill, an elongated palatinum, an upward positioned cranio-facial hinge and upward positioned orbits (Fig. 10). The correlation between the scores of the second PLS_*sep *_vector was low (r = 0.518) and therefore, the corresponding shape changes are not visualized.

## Discussion

In avian morphological studies, geometric morphometric methods are not commonly applied and when they are, they encounter particular difficulties (compare [36-38]). As bony junctions on avian skulls are not externally visible, there are only few anatomical landmarks of type I [31]. Instead of relying on the scarce anatomical landmarks, here we quantified the skull using the method of semilandmarks. These semilandmarks can be placed on "forms without landmarks" [26,28] and make it possible to incorporate curvature information in coordinate-based morphometrics.

Because the shape of the bill, particularly its length, is highly variable [36,37], landmarks placed on the tip of the bill create a "pinocchio effect". If two crania were completely identical in shape, but had bills of different lengths, then the least squares properties of the Procrustes registration would "create" shape differences on every landmark. This methodological artifact, however, only affects the Procrustes superimposition, not the thin-plate spline. Because semilandmarks (in their bending energy version) are built upon the thin-plate spline algebra and the visualizations are computed as thin-plate spline deformations, they are not affected by registration problems.

In this study we adjusted GPA by using centroid size calculated only from landmarks and semilandmarks placed on the cranium and the antorbital fenestra (see Methods). For similar reasons van der Meij [37] did not include landmarks placed on the bill-tip in GPA and added these landmarks to the fitted data after applying the same scaling, rotation and translation as for the other landmarks. Marugan-Lobon and Buscalioni [36] ran separate GPAs, one including the landmark on the bill-tip and one without.

### Species differences

Major shape variation occurs at the bill (PC1), in the position of the orbits and foramen magnum (PC1 and PC2) and in the angle between bill and cranium (PC2). These results are consistent with quantitative studies on avian skull morphology, which also report of major variation at the bill, in the position of the foramen magnum and in the angle between bill and cranium [36,37].

According to scores in PC 1, the common raven has the longest, widest, most curved bill; the longest, widest and most flattened cranium; the most upward positioned foramen magnum; and the most sidewise oriented orbits, followed by the rook, hooded crow, black-billed magpie and Eurasian jay (Fig. 2, Fig. 3a). PC 1 correlates positively with log centroid size and in addition, the first eigenvector is highly correlated to the vector of regression slopes returned by a multivariate linear regression of the Procrustes shape coordinates on log centroid size. Although, these results suggest that shape changes with increasing centroid size are similar across species, the within-species multivariate regression of the Procrustes shape coordinates on log centroid size indicates that the allometric relationships differ markedly between species (compare [39], Fig. 4). Despite the fact that our intraspecific sample is small and consists only of adult, unsexed corvids, it seems likely that shape changes observed between species are not only due to differences in size, but can also be assumed to vary according to differences in ecology. In birds, it is known that the shape allometry of the bill is very variable between species (e.g. [40]). Furthermore it can even vary within species, e.g. due to sexual dimorphism [39]. These allometric differences are often associated with ecological factors such as foraging behavior [41,42].

PC 2 separates the small corvid species (jackdaw, black-billed magpie and Eurasian jay, Fig. 2) and does not correlate with log centroid size. Hence, the corresponding shape changes are not allometric. The Eurasian jay with the highest scores in PC 2 has the highest angle between bill and cranium, most curved and thinnest bill and cranium, most downward positioned foramen magnum and most upward and sidewise oriented orbits, followed by black-billed magpie and jackdaw (Fig. 3b). Bigger corvid species (hooded crow, rook and common raven) overlap in PC 2 and have scores approximately equal to black-billed magpie (Fig. 2).

### Morphological integration

Partial least squares is a relatively novel technique to study integration and we have employed the most recent algorithm that takes the scaling of the PLS loading vectors into account (compare [32,34,43]). The results of the PLS analysis showed that none of the dimensions displayed a clear distinction between species. Instead, the pattern of covariation between blocks is shared across all species and thus might be due to similar constraints (ecological, developmental and/or biomechanical) between species.

Most of the covariation in the first dimension of the PLS_*whole *_describes differences in bill- and cranium-length and in the orientation of the orbits (Fig. 7). Thus, it is seems likely that PLS_*whole *_1 describes shape differences that are due to individual differences in size. The second dimension of PLS_*whole *_and the first dimension of PLS_*sep *_indicate that most of the covariation occurs at the cranio-facial hinge and results in a change in the angulation between bill and cranium (Fig. 8, Fig. 10). While this difference in angulation exhibited by PLS_*whole *_2 is due to a rotation of the bill and cranium in opposite directions (Fig. 8), in PLS_*sep *_1 (and PLS_*whole *_3) it is due to a local effect, i.e. to a change in the position of the cranio-facial hinge (Fig. 10). In corvids, the cranio-facial hinge is known to be a bending zone, a clearly recognizable area of thin bone, between the movable upper bill and the cranium [20,44]. Thus, it is not surprising that variation in the morphological integration between individuals occur in this specialized zone (compare [45,46]).

Both, the cranio-facial hinge and the palatinum are part of the mechanism that enables corvids to move the upper bill (rhynchokinetics, [20,44]). Hence, variation in the relative length (PLS_*sep *_1) and in the relative position (PLS_*whole *_2) of the palatinum are likely to influence the properties of the rhynchokinetics (e.g. opening angle, bite force).

### Functional implications

#### Bill

Previous studies on the foraging behavior of corvids suggest that the studied species vary in the frequency with which they use their repertoire of foraging techniques [4-8]. Rooks are most frequently observed probing followed by hooded crow, black-billed magpie and jackdaw (and vice versa in pecking [4-8]). These differences are expected to covary with bill-length [15-18] and -curvature, because a curved bill is thought to allow inspection of a greater volume of sediment than a straight bill of equal length [17]. Hence, our results suggest that bill-length and -curvature is progressively increased from jackdaw, to Eurasian jay, to black-billed magpie, to hooded crow and to rook and thus meet our predictions stated above.

These differences become even more apparent, when feeding on carcasses is considered as probing as well. The common raven, which has the longest and most curved bill of the studied corvid species, feed primarily on carcasses, which are, when unopened, accessed through orifices. Thus, a long bill might be beneficial to intrude deeply into the orifices and in addition a heavily curved bill enhances the ability to rip meet apart [1,9,10]. Although there are no comparative studies on the foraging behavior of Eurasian jays that we know of, our results on bill-length suggest that they probe more frequently than jackdaws and less frequently than black-billed magpie (and vice versa in pecking).

The frequency with which corvid species turn objects is not as clear as for probing and pecking [4-7]. All studies in which jackdaws were observed, reported that jackdaws show the highest frequency in turning objects compared with rook [4,6], carrion crow and black-billed magpie [4]. Our results indicate that jackdaws have the steepest angle between bill and cranium. It seems likely that this feature is beneficial to turn objects, because an upward positioned bill-tip relative to the cranium might facilitate shoveling movements with the bill, compared with a more downward positioned bill-tip as in e.g. Eurasian jay.

#### Orbits

Birds that frequently probe are also assumed to require a smaller binocular field as opposed to birds that frequently peck and turn objects, because the frontal binocular field is thought to guide the bill towards food objects [22,23]. It is likely that changes in orbit position indicate changes in eye position and in addition, several authors assumed that orbit convergence is associated with the degree of the binocular field overlap [23-25]. When assuming similar photoreceptor densities (compare [22,23,47]), our results indicate that common ravens have the narrowest binocular field, followed by rook, hooded crow, Eurasian jay, black-billed magpie and jackdaw and thus would meet the prediction that probing is associated with a smaller binocular field compared to pecking.

Another important aspect of the frontal binocular field in birds is the horizontal projection of the bill-tip. Species that handle food objects between their mandibles have been reported to be able to observe their own bill-tip [48-51]. It has been hypothesized that this ability is concordant with an increased angle between bill and cranium and/or upward positioned eyes, which result in the bill aperture pointing toward the mid-point of the eyes [16,19,52]. Although, it is not known whether corvids are able to observe their own bill-tip, it seems reasonable that at least the Eurasian jay should have the capability for two reasons. First, the Eurasian jay is known to handle food items, i.e. acorns, between its mandibles [9,53] and second, the jay displays morphological adjustments which results in the bill aperture pointing toward the mid point of the eyes (Fig. 3b). Consequently the first PLS_*whole *_vectors, which also indicate major covariation between bill-length and orbit orientation, might be considered as an adjustment of the frontal binocular field relative to the bill-tip, so that the horizontal projection of the bill-tip within the binocular field does not change between individuals with different bill-length.

Another likely explanation for the increased angle between bill and cranium and upward positioned eyes found in Eurasian Jays, when compared with the other corvid-species, might be a more downward projection of the bill-tip within the binocular field and an increased vertical extent of the binocular field. These features might be beneficial when gleaning for caterpillar larvae at the lower surface of leaves as reported in Owen [54], because it would allow Eurasian jays to gather visual information above head, while the head is held horizontal.

On the other hand, the decreased angle between bill and cranium and downward positioned eyes found in jackdaws would result in a more upward projection of the bill-tip within the binocular field and might allow jackdaws to gather visual information below the head when the head is held horizontal. This ability might be beneficial when turning objects and searching for food under surface litter, which is a frequent foraging strategy of jackdaws [4,6]. Accordingly, the second PLS_*whole *_and first PLS_*sep *_vectors indicate major covariation between blocks in the angle between bill and cranium, so that differences in the angulation might result in differences in the vertical projection of the bill-tip within the binocular field.

#### Foramen magnum

Species with high scores on PC 1 (e.g. common raven) and low scores on PC 2 (e.g. jackdaw) are characterized by an upward positioned foramen magnum, compared to Eurasian jay and black-billed magpie. The within-species multivariate regression of size on shape showed a strong relationship of size on the position of the foramen magnum in hooded crow, black-billed magpie and jackdaw, but either no or a weak relationship in common raven, Eurasian jay and rook (compare Fig. 4). The position of the foramen magnum might be associated with head posture (compare [36,55], exemplified in Fig. 8). Hence, species with an upward positioned foramen magnum, e.g. common raven and jackdaw, might be characterized by a more horizontal head posture and according to PCA have also a more flattened cranium, compared to Eurasian jay and black-billed magpie. Differences in head posture and height of the cranium might be attributed to constraints in sustained flight. Species with a horizontal head posture and a flattened cranium might experience a reduced drag in flight, compared to species with a vertical head posture and increased cranial height [56]. Furthermore, differences in head posture between the studied corvid-species seem to covary with wingtip-shape [57], which have been reported to influence flight ability [58]. Thus, the *Corvus*-species are characterized by pointed wingtips and possibly by a horizontal head posture and hence, might show an increased flight ability, compared to Eurasian jay and black-billed magpie [9,10].

As the number of studied species is small, further research is necessary to evaluate the relationship between the position of the foramen magnum and head posture and their covariation to other morphological traits.

## Conclusion

In this study we compared skull morphology and the integration between the bill and cranium of six corvid species by means of three-dimensional geometric morphometrics of computed tomography scans. Our results indicate that pronounced shape differences occur between the studied corvid species. Although most of the shape variation correlates with size, these shape differences cannot be attributed only to allometry, because the allometric components differ markedly between species. Thus, shape differences between species can also be considered to result from differences in ecology, especially because skull morphology covaries with foraging mode. Increasing bill-length, bill-curvature and sidewise orientation of the eyes is associated with an increase in the observed frequency in probing (vice versa in pecking). Hence, the frequency of probing, bill-length, bill-curvature and sidewise orientation of the eyes is progressively increased from jackdaw, to Eurasian jay, to black-billed magpie, to hooded crow, to rook and to common raven (when feeding on carcasses is considered as probing). With regards to morphological integration, our results suggest that most of the covariation between bill and cranium is due to differences in the topography of the binocular fields and the projection of the bill-tip therein, indicating the importance of visual fields in the foraging ecology of corvids. Further research, especially the morphological integration between bill, cranium and components that are involved in the rhynchokinetics of birds, is of great interest to study functional and species wide trends.

## Competing interests

The authors declare that they have no competing interests.

## Authors' contributions

CK designed the study, analyzed the data and drafted the manuscript. KA placed landmarks and semilandmarks. PG participated and supervised the data analysis. SF and FB supervised the study and were also involved in drafting the manuscript. All authors read and approved the final manuscript.
